# Bis(2,9-dimethyl-1,10-phenanthrolin-1-ium) hydrogen (*S*,*S*)-tartrate nona­hydrate

**DOI:** 10.1107/S160053681005110X

**Published:** 2010-12-11

**Authors:** Zohreh Derikvand, Marilyn M. Olmstead

**Affiliations:** a Young Researchers Club, Islamic Azad University, Khorramabad Branch, Khorramabad, Iran; b Department of Chemistry, University of California, One Shields Avenue, Davis, CA 95616-5292, USA

## Abstract

The asymmetric unit of the title compound, 2C_14_H_13_N_2_^+^·2C_4_H_5_O_6_^−^·9H_2_O, contains two cations and two anions in addition to nine mol­ecules of water. Each of the hydrogen tartrate anions is hydrogen bonded to itself by translation along [100] in a head-to-tail fashion *via* a short hydrogen bond with donor–acceptor distances of 2.473 (4) and 2.496 (4) Å. A large number of inter­molecular O–H⋯O, N—H⋯O and C–H⋯O hydrogen-bonding inter­actions, as well as π–π stacking [centroid–centroid distances in the range 3.642 (3) to 3.866 (3) Å], play an important role in the crystal structure.

## Related literature

For proton-transfer structures of tartaric acid, see: Bai *et al.* (2005 ▶); Derikvand & Olmstead (2010 ▶); Paixão *et al.* (1999 ▶); Ryttersgaard & Larsen (2003 ▶); Smith *et al.* (2006 ▶); Su *et al.* (2009 ▶); Suresh *et al.* (2006 ▶); Wang *et al.* (2008 ▶); Zhang *et al.* (2006 ▶).
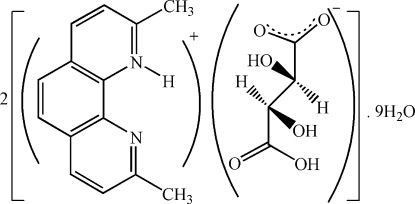


## Experimental

### 

#### Crystal data

2C_14_H_13_N_2_^+^·2C_4_H_5_O_6_^−^·9H_2_O
*M* *_r_* = 878.83
Orthorhombic, 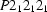

*a* = 7.0927 (5) Å
*b* = 23.3998 (15) Å
*c* = 24.9335 (16) Å
*V* = 4138.2 (5) Å^3^
*Z* = 4
Mo *K*α radiation
μ = 0.12 mm^−1^
*T* = 90 K
0.53 × 0.06 × 0.05 mm

#### Data collection

Bruker APEXII diffractometer
Absorption correction: multi-scan (*SADABS*; Sheldrick, 1996 ▶) *T*_min_ = 0.975, *T*_max_ = 0.994
49446 measured reflections
7023 independent reflections
6177 reflections with *I* > 2σ(*I*)
*R*_int_ = 0.097

#### Refinement

*R*[*F* ^2^ > 2σ(*F* ^2^)] = 0.090
*wR*(*F* ^2^) = 0.173
*S* = 1.25
7023 reflections
611 parameters
111 restraints
H atoms treated by a mixture of independent and constrained refinement
Δρ_max_ = 0.47 e Å^−3^
Δρ_min_ = −0.46 e Å^−3^

### 

Data collection: *APEX2* (Bruker, 2009 ▶); cell refinement: *SAINT* (Bruker, 2009 ▶); data reduction: *SAINT*; program(s) used to solve structure: *SHELXS97* (Sheldrick, 2008 ▶); program(s) used to refine structure: *SHELXL97* (Sheldrick, 2008 ▶); molecular graphics: *SHELXTL* (Sheldrick, 2008 ▶); software used to prepare material for publication: *SHELXL97*.

## Supplementary Material

Crystal structure: contains datablocks I, global. DOI: 10.1107/S160053681005110X/hg2765sup1.cif

Structure factors: contains datablocks I. DOI: 10.1107/S160053681005110X/hg2765Isup2.hkl

Additional supplementary materials:  crystallographic information; 3D view; checkCIF report

## Figures and Tables

**Table 1:** Hydrogen-bond geometry (Å, °)

*D*—H⋯*A*	*D*—H	H⋯*A*	*D*⋯*A*	*D*—H⋯*A*
N2—H2*A*⋯O1*W*	0.88	1.97	2.820 (5)	162
N4—H4*C*⋯O7*W*	0.88	1.98	2.814 (5)	159
O4—H4⋯O6*W*	0.84	1.80	2.633 (5)	175
O9—H9*C*⋯O5*W*	0.84	1.83	2.673 (4)	177
O10—H10*A*⋯O4*W*	0.84	1.79	2.627 (5)	172
O2*W*—H2*C*⋯O11	0.86 (4)	1.97 (2)	2.783 (4)	157 (4)
O3*W*—H3*C*⋯O5	0.87 (4)	1.95 (2)	2.798 (5)	168 (5)
O2*W*—H2*D*⋯O8*W*	0.88	2.42	2.903 (5)	115
O5*W*—H5*A*⋯O3	0.87 (4)	2.04 (3)	2.827 (4)	151 (5)
O5*W*—H5*A*⋯O2	0.87 (4)	2.35 (4)	3.020 (4)	135 (5)
O8*W*—H8*B*⋯O2*W*	0.87 (2)	2.03 (2)	2.903 (5)	178 (5)
O9*W*—H9*A*⋯O2	0.86 (4)	1.89 (2)	2.744 (4)	175 (5)
O9*W*—H9*B*⋯O9	0.87 (4)	2.08 (4)	2.849 (4)	147 (5)
O9*W*—H9*B*⋯O8	0.87 (4)	2.26 (4)	2.958 (4)	137 (5)
O1—H1⋯O5^i^	0.99 (6)	1.49 (6)	2.473 (4)	172 (5)
O7—H7⋯O11^i^	0.91 (6)	1.60 (6)	2.496 (4)	167 (5)
O4*W*—H4*B*⋯O12^i^	0.87 (2)	1.85 (2)	2.720 (5)	178 (6)
O6*W*—H6*B*⋯O6^i^	0.86 (3)	1.93 (2)	2.776 (5)	167 (5)
O3—H3*B*⋯O9*W*^ii^	0.84	1.81	2.647 (5)	173
O5*W*—H5*B*⋯O8^ii^	0.87 (4)	1.91 (2)	2.761 (4)	168 (5)
O1*W*—H1*A*⋯O2*W*^iii^	0.87 (4)	2.22 (3)	3.062 (5)	161 (5)
O1*W*—H1*B*⋯O5*W*^iii^	0.87 (4)	1.96 (2)	2.811 (5)	168 (6)
O3*W*—H3*D*⋯O8*W*^iii^	0.87 (4)	2.02 (2)	2.876 (5)	168 (5)
O4*W*—H4*A*⋯O10^iv^	0.87 (4)	1.99 (3)	2.815 (4)	160 (5)
O4*W*—H4*A*⋯O12^iv^	0.87 (4)	2.40 (4)	2.998 (4)	127 (4)
O6*W*—H6*A*⋯O4^v^	0.87 (4)	2.15 (4)	2.892 (4)	143 (5)
O6*W*—H6*A*⋯O6^v^	0.87 (4)	2.19 (4)	2.909 (5)	140 (5)
O7*W*—H7*A*⋯O3*W*^vi^	0.87 (4)	2.17 (3)	2.988 (5)	156 (5)
O7*W*—H7*B*⋯O9*W*^vii^	0.87 (4)	2.02 (2)	2.869 (5)	165 (5)
O8*W*—H8*A*⋯O1^vii^	0.87 (4)	2.06 (2)	2.926 (5)	170 (5)

Symmetry codes: (i) 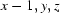; (ii) 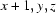; (iii) 
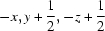
; (iv) 
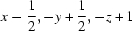
; (v) 
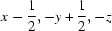
; (vi) 
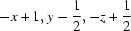
; (vii) 
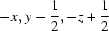
.

**Fig. 1.**
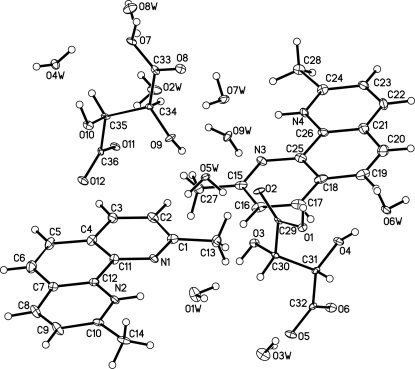
A drawing of the asymmetric unit of the title compound. Thermal ellipsoids are drawn at the 50% probability level.

**Fig. 2.**
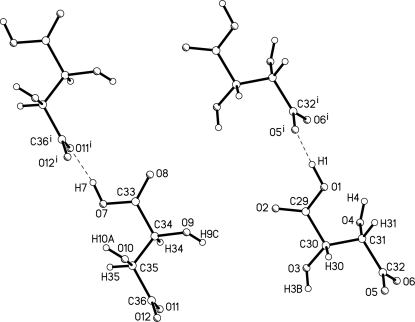
The hydrogen bonding interactions that support the strong hydrogen bonding between tartrate anions (symmetry code: i = x - 1, y, z).

**Fig. 3.**
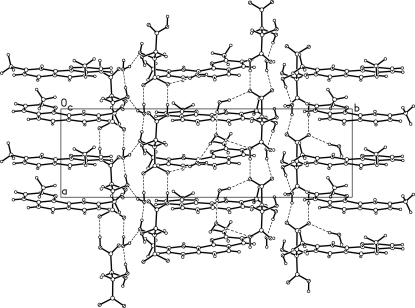
Crystal packing diagram as viewed down c.

## supplementary crystallographic information

### Comment

We recently reported the structure of the trihydrate of a salt formed by proton transfer from D-tartaric acid to phenanthroline (Derikvand & Olmstead, 2010). An interesting feature of this structure was the existence of a short hydrogen bond between adjacent tartrate anions. There are many other proton transfer structures of tartaric acid, for example, (Paixão *et al.*, 1999; Ryttersgaard & Larsen, 2003; Bai *et al.*, 2005; Zhang *et al.*, 2006; Smith, *et al.*, 2006; Suresh *et al.*, 2006; Wang *et al.*, 2008; Su *et al.*, 2009), some of which feature similar short hydrogen bonds. The title structure contains two protonated cations of 2,9-dimethyl-1,10-phenanthroline (neocuproine), two anions of mono-deprotonated D-tartaric acid and nine water molecules. As shown in Fig. 1, one of the protons of the tartaric acid carboxylic groups has been transferred to one of the nitrogen atoms of the 2,9-dimethyl-1,10-phenanthroline molecule. The structure reveals a pattern similar to that seen in the previous structure of (phen)(D-tartrate)^.^3H_2_O. In particular, the carboxylic acid group at one end of the tartrate anion is hydrogen bonded to the deprotonated carboxylic acid group of an adjacent tartrate anion in a linear, head-to-tail fashion. Each of the two tartrate anions in the asymmetric unit displays this kind of hydrogen bond (Fig. 2). These hydrogen bonds are short (O···O distances of 2.473 (4) Å and 2.496 (4) Å), and are propagated by unit translations of the anions along the a-direction. In addition, the O—H distance is longer, the H···O distance is shorter and the O—H···O angle is more linear than other O—H···O interactions in the structure. A large number of additional hydrogen bonding interactions are also present. It is notable that crystal growth along the a-direction is clearly preferred. In the crystal used for data collection, the longest dimension, 0.53 mm, corresponds to the [100] direction. The two shorter dimensions, 0.05 mm and 0.06 mm, correspond to [010] and [001], respectively. Details of the O–H···O, N–H···O and C–H···O hydrogen bonds are given in Table 1. As was seen in the structure containing phen, the cationic dmpH^+^ units are stacked together *via*π–π interactions. The centroid-centroid distances fall in the range 3.642 (3) Å to 3.866 (3) Å. The overall packing of the structure is depicted in Fig. 3.

### Experimental

To an aqueous solution of D-tartaric acid (75 mg, 1 mmol) in water (10 ml) was added a solution of 2,9-dimethyl-1,10-phenanthroline (100 mg, 1 mmol) in methanol (10 ml) in a 1:1 molar ratio. The solution was stirred for 1 h. By slow evaporation of the solvent at room temperature, colorless needles were obtained after 1 week.

### Refinement

Hydrogen atoms H1 and H7 were allowed to freely refine with isotropic thermal parameters tied to 1.2 times the equivalent isotropic values of their parent O atoms. Water hydrogen atoms were located in a difference Fourier map and subsequently refined with restraints of 0.87 (2) for O—H distances and 1.34 (4) for O···O distances. N—H distances were restrained to 0.88 Å, hydroxyl O—H to 0.84 Å, and C—H to 0.98 Å using a riding model and U(iso) = 1.2 U(eq). The carbon atoms C29 to C36 were restrained to ISOR 0.004. In addition, the positional parameters of H2D were fixed due to disorder involving O2W and O8W that could not be resolved. In the absence of significant anomalous dispersion, Friedel opposites were merged in the final cycles of refinement.

### Crystal data

**Table:**

2C_14_H_13_N_2_^+^·2C_4_H_5_O_6_^−^·9H_2_O	*D*_x_ = 1.411 Mg m^−^^3^
*M**_r_* = 878.83	Melting point: 523 K
Orthorhombic, *P*2_1_2_1_2_1_	Mo *K*α radiation, λ = 0.71073 Å
Hall symbol: P 2ac 2ab	Cell parameters from 7481 reflections
*a* = 7.0927 (5) Å	θ = 3.0–30.5°
*b* = 23.3998 (15) Å	µ = 0.12 mm^−^^1^
*c* = 24.9335 (16) Å	*T* = 90 K
*V* = 4138.2 (5) Å^3^	Needle, colorless
*Z* = 4	0.53 × 0.06 × 0.05 mm
*F*(000) = 1864	

### Data collection

**Table:**

Bruker APEXII diffractometer	7023 independent reflections
Radiation source: fine-focus sealed tube	6177 reflections with *I* > 2σ(*I*)
graphite	*R*_int_ = 0.097
Detector resolution: 8.3 pixels mm^-1^	θ_max_ = 30.5°, θ_min_ = 2.6°
ω scans	*h* = −10→10
Absorption correction: multi-scan (*SADABS*; Sheldrick, 1996)	*k* = −32→33
*T*_min_ = 0.975, *T*_max_ = 0.994	*l* = −35→35
49446 measured reflections	

### Refinement

**Table:**

Refinement on *F*^2^	Primary atom site location: structure-invariant direct methods
Least-squares matrix: full	Secondary atom site location: difference Fourier map
*R*[*F*^2^ > 2σ(*F*^2^)] = 0.090	Hydrogen site location: inferred from neighbouring sites
*wR*(*F*^2^) = 0.173	H atoms treated by a mixture of independent and constrained refinement
*S* = 1.25	*w* = 1/[σ^2^(*F*_o_^2^) + (0.0387*P*)^2^ + 8.4022*P*] where *P* = (*F*_o_^2^ + 2*F*_c_^2^)/3
7023 reflections	(Δ/σ)_max_ = 0.001
611 parameters	Δρ_max_ = 0.47 e Å^−^^3^
111 restraints	Δρ_min_ = −0.46 e Å^−^^3^

### Special details

**Table:**

Refinement. Refinement of *F*^2^ against ALL reflections. The weighted *R*-factor *wR* and goodness of fit *S* are based on *F*^2^, conventional *R*-factors *R* are based on *F*, with *F* set to zero for negative *F*^2^. The threshold expression of *F*^2^ > σ(*F*^2^) is used only for calculating *R*-factors(gt) *etc*. and is not relevant to the choice of reflections for refinement. *R*-factors based on *F*^2^ are statistically about twice as large as those based on *F*, and *R*- factors based on ALL data will be even larger.

### Fractional atomic coordinates and isotropic or equivalent isotropic displacement parameters (Å^2^)

**Table:**

	*x*	*y*	*z*	*U*_iso_*/*U*_eq_	
N1	0.0327 (5)	0.46527 (16)	0.33329 (14)	0.0095 (7)	
N2	0.0442 (6)	0.56743 (15)	0.38606 (15)	0.0099 (7)	
H2A	0.0154	0.5648	0.3518	0.012*	
C1	0.0288 (7)	0.41562 (18)	0.30782 (18)	0.0117 (8)	
C2	0.0453 (7)	0.36264 (18)	0.33514 (18)	0.0113 (8)	
H2	0.0373	0.3279	0.3156	0.014*	
C3	0.0728 (7)	0.36145 (19)	0.38948 (18)	0.0123 (9)	
H3	0.0835	0.3262	0.4080	0.015*	
C4	0.0850 (7)	0.41442 (18)	0.41757 (18)	0.0109 (8)	
C5	0.1207 (7)	0.4176 (2)	0.47412 (18)	0.0134 (9)	
H5	0.1305	0.3834	0.4946	0.016*	
C6	0.1406 (7)	0.4690 (2)	0.49855 (19)	0.0163 (9)	
H6	0.1709	0.4705	0.5356	0.020*	
C7	0.1163 (7)	0.52117 (19)	0.46895 (18)	0.0117 (8)	
C8	0.1368 (8)	0.5760 (2)	0.49209 (19)	0.0172 (10)	
H8	0.1722	0.5795	0.5287	0.021*	
C9	0.1059 (7)	0.62418 (19)	0.46203 (19)	0.0154 (9)	
H9	0.1173	0.6608	0.4781	0.018*	
C10	0.0575 (6)	0.61955 (18)	0.40778 (18)	0.0105 (8)	
C11	0.0611 (7)	0.46398 (18)	0.38698 (17)	0.0101 (8)	
C12	0.0728 (6)	0.51851 (18)	0.41399 (17)	0.0098 (8)	
C13	0.0119 (7)	0.4179 (2)	0.24814 (18)	0.0138 (9)	
H13A	−0.0464	0.4542	0.2376	0.021*	
H13B	−0.0664	0.3861	0.2357	0.021*	
H13C	0.1376	0.4150	0.2320	0.021*	
C14	0.0211 (7)	0.67108 (18)	0.37334 (19)	0.0125 (9)	
H14A	0.0576	0.6627	0.3363	0.019*	
H14B	0.0953	0.7034	0.3867	0.019*	
H14C	−0.1132	0.6807	0.3746	0.019*	
N3	0.5130 (5)	0.01878 (15)	0.17341 (15)	0.0100 (7)	
N4	0.4445 (6)	−0.07881 (15)	0.11789 (14)	0.0092 (7)	
H4C	0.4710	−0.0784	0.1524	0.011*	
C15	0.5497 (7)	0.06682 (19)	0.20033 (19)	0.0124 (9)	
C16	0.5333 (7)	0.12188 (18)	0.1758 (2)	0.0143 (9)	
H16	0.5585	0.1553	0.1962	0.017*	
C17	0.4817 (7)	0.1269 (2)	0.1230 (2)	0.0151 (9)	
H17	0.4715	0.1633	0.1065	0.018*	
C18	0.4442 (7)	0.07635 (19)	0.09370 (19)	0.0135 (9)	
C19	0.3935 (7)	0.0763 (2)	0.0377 (2)	0.0168 (10)	
H19	0.3816	0.1117	0.0193	0.020*	
C20	0.3627 (8)	0.0273 (2)	0.0110 (2)	0.0195 (10)	
H20	0.3306	0.0286	−0.0260	0.023*	
C21	0.3776 (7)	−0.0270 (2)	0.03751 (19)	0.0155 (9)	
C22	0.3541 (7)	−0.0806 (2)	0.01155 (18)	0.0150 (9)	
H22	0.3215	−0.0817	−0.0254	0.018*	
C23	0.3776 (7)	−0.13030 (19)	0.03872 (18)	0.0125 (9)	
H23	0.3621	−0.1658	0.0207	0.015*	
C24	0.4246 (7)	−0.12946 (19)	0.09344 (18)	0.0115 (8)	
C25	0.4603 (7)	0.02428 (19)	0.12140 (18)	0.0120 (8)	
C26	0.4256 (7)	−0.02800 (19)	0.09198 (18)	0.0115 (8)	
C27	0.6064 (8)	0.0606 (2)	0.25742 (19)	0.0164 (10)	
H27A	0.6325	0.0204	0.2652	0.025*	
H27B	0.5042	0.0741	0.2806	0.025*	
H27C	0.7201	0.0834	0.2641	0.025*	
C28	0.4518 (7)	−0.18329 (17)	0.12542 (18)	0.0116 (8)	
H28A	0.5132	−0.1741	0.1596	0.017*	
H28B	0.5311	−0.2099	0.1051	0.017*	
H28C	0.3290	−0.2009	0.1324	0.017*	
O1	0.0522 (5)	0.35066 (13)	0.12922 (12)	0.0088 (6)	
H1	−0.088 (8)	0.353 (2)	0.129 (2)	0.011*	
O2	0.0346 (4)	0.28017 (13)	0.18981 (13)	0.0101 (6)	
O3	0.4033 (5)	0.25951 (12)	0.18514 (12)	0.0094 (6)	
H3B	0.5020	0.2671	0.2023	0.011*	
O4	0.3405 (5)	0.26187 (13)	0.07110 (13)	0.0101 (6)	
H4	0.2505	0.2719	0.0513	0.012*	
O5	0.7038 (4)	0.35075 (13)	0.12500 (13)	0.0096 (6)	
O6	0.7107 (5)	0.27534 (13)	0.07009 (13)	0.0110 (6)	
C29	0.1247 (6)	0.31162 (17)	0.16055 (16)	0.0068 (8)	
C30	0.3399 (6)	0.30930 (18)	0.15785 (16)	0.0059 (7)	
H30	0.3918	0.3436	0.1766	0.007*	
C31	0.4082 (6)	0.30995 (18)	0.09990 (16)	0.0068 (8)	
H31	0.3597	0.3454	0.0822	0.008*	
C32	0.6237 (6)	0.31068 (17)	0.09741 (16)	0.0062 (7)	
O7	−0.4298 (4)	0.13513 (13)	0.38098 (13)	0.0088 (6)	
H7	−0.557 (9)	0.132 (2)	0.377 (2)	0.011*	
O8	−0.4525 (5)	0.19870 (13)	0.31384 (13)	0.0102 (6)	
O9	−0.0879 (5)	0.22177 (13)	0.31636 (12)	0.0101 (6)	
H9C	0.0058	0.2126	0.2976	0.012*	
O10	−0.1366 (5)	0.22847 (13)	0.43072 (13)	0.0096 (6)	
H10A	−0.2398	0.2219	0.4460	0.012*	
O11	0.2185 (4)	0.13303 (13)	0.38332 (12)	0.0077 (6)	
O12	0.2307 (5)	0.21666 (13)	0.42670 (13)	0.0112 (6)	
C33	−0.3603 (6)	0.17098 (17)	0.34588 (17)	0.0073 (8)	
C34	−0.1458 (6)	0.17432 (17)	0.34727 (17)	0.0061 (7)	
H34	−0.0937	0.1389	0.3305	0.007*	
C35	−0.0721 (6)	0.17825 (17)	0.40533 (17)	0.0059 (7)	
H35	−0.1189	0.1444	0.4259	0.007*	
C36	0.1436 (6)	0.17735 (18)	0.40528 (16)	0.0066 (8)	
O1W	−0.0728 (6)	0.58367 (14)	0.27904 (14)	0.0177 (7)	
H1A	−0.076 (9)	0.5612 (18)	0.2511 (15)	0.021*	
H1B	−0.132 (8)	0.6140 (15)	0.268 (2)	0.021*	
O2W	0.1114 (5)	0.03182 (14)	0.33319 (14)	0.0173 (7)	
H2C	0.153 (8)	0.0565 (14)	0.3558 (16)	0.021*	
H2D	0.1089	0.0003	0.3521	0.021*	
O3W	0.5757 (5)	0.45505 (14)	0.16602 (14)	0.0161 (7)	
H3C	0.599 (8)	0.4224 (14)	0.151 (2)	0.019*	
H3D	0.466 (5)	0.464 (2)	0.153 (2)	0.019*	
O4W	−0.4478 (5)	0.21368 (13)	0.48609 (12)	0.0100 (6)	
H4A	−0.479 (7)	0.2357 (18)	0.5126 (14)	0.012*	
H4B	−0.550 (5)	0.215 (2)	0.4667 (17)	0.012*	
O5W	0.2110 (5)	0.18871 (13)	0.25895 (12)	0.0090 (6)	
H5A	0.229 (7)	0.2143 (17)	0.2344 (14)	0.011*	
H5B	0.307 (5)	0.195 (2)	0.2797 (17)	0.011*	
O6W	0.0447 (5)	0.29367 (14)	0.01471 (13)	0.0112 (6)	
H6A	0.035 (8)	0.2697 (17)	−0.0118 (14)	0.013*	
H6B	−0.064 (4)	0.293 (2)	0.0301 (19)	0.013*	
O7W	0.4713 (5)	−0.10319 (14)	0.22815 (13)	0.0143 (7)	
H7A	0.453 (8)	−0.0775 (16)	0.2527 (17)	0.017*	
H7B	0.407 (7)	−0.1320 (15)	0.240 (2)	0.017*	
O8W	−0.2016 (5)	−0.03301 (14)	0.37713 (14)	0.0153 (7)	
H8A	−0.146 (7)	−0.0662 (12)	0.378 (2)	0.018*	
H8B	−0.108 (5)	−0.0129 (18)	0.365 (2)	0.018*	
O9W	−0.3015 (5)	0.28971 (14)	0.24357 (12)	0.0105 (6)	
H9A	−0.200 (5)	0.286 (2)	0.2251 (18)	0.013*	
H9B	−0.283 (8)	0.2656 (18)	0.2694 (14)	0.013*	

### Atomic displacement parameters (Å^2^)

**Table:**

	*U*^11^	*U*^22^	*U*^33^	*U*^12^	*U*^13^	*U*^23^
N1	0.0071 (16)	0.0114 (17)	0.0099 (16)	−0.0012 (14)	−0.0031 (14)	−0.0001 (13)
N2	0.0111 (17)	0.0084 (17)	0.0102 (16)	−0.0026 (14)	−0.0035 (15)	−0.0004 (13)
C1	0.012 (2)	0.009 (2)	0.015 (2)	0.0000 (17)	0.0034 (17)	−0.0036 (16)
C2	0.014 (2)	0.0051 (18)	0.015 (2)	0.0008 (16)	−0.0018 (18)	−0.0039 (15)
C3	0.013 (2)	0.011 (2)	0.0127 (19)	−0.0005 (17)	−0.0035 (18)	0.0029 (16)
C4	0.010 (2)	0.011 (2)	0.0119 (19)	0.0030 (17)	0.0004 (16)	0.0020 (15)
C5	0.015 (2)	0.014 (2)	0.012 (2)	0.0048 (18)	−0.0010 (18)	0.0074 (16)
C6	0.018 (2)	0.019 (2)	0.012 (2)	0.001 (2)	−0.0013 (19)	0.0028 (18)
C7	0.010 (2)	0.013 (2)	0.0119 (19)	−0.0019 (17)	−0.0003 (17)	0.0003 (16)
C8	0.019 (2)	0.019 (2)	0.013 (2)	−0.001 (2)	−0.0055 (19)	−0.0060 (18)
C9	0.019 (2)	0.010 (2)	0.017 (2)	0.0022 (18)	−0.005 (2)	−0.0097 (17)
C10	0.0056 (19)	0.011 (2)	0.015 (2)	−0.0015 (16)	−0.0013 (17)	0.0000 (16)
C11	0.012 (2)	0.0079 (19)	0.0100 (19)	0.0017 (16)	0.0003 (17)	−0.0007 (15)
C12	0.007 (2)	0.010 (2)	0.0118 (19)	−0.0013 (16)	0.0012 (16)	−0.0010 (15)
C13	0.021 (2)	0.011 (2)	0.0097 (18)	0.0004 (18)	0.0034 (17)	−0.0036 (16)
C14	0.008 (2)	0.0073 (19)	0.022 (2)	−0.0009 (16)	−0.0018 (18)	−0.0030 (17)
N3	0.0077 (17)	0.0094 (17)	0.0130 (17)	0.0006 (13)	−0.0016 (14)	−0.0025 (14)
N4	0.0122 (18)	0.0065 (16)	0.0089 (16)	−0.0011 (14)	−0.0017 (15)	−0.0023 (13)
C15	0.010 (2)	0.011 (2)	0.017 (2)	−0.0013 (17)	0.0069 (18)	−0.0055 (16)
C16	0.015 (2)	0.0040 (19)	0.024 (2)	0.0016 (16)	0.011 (2)	−0.0054 (16)
C17	0.009 (2)	0.009 (2)	0.027 (2)	0.0023 (16)	0.0029 (19)	−0.0036 (18)
C18	0.011 (2)	0.009 (2)	0.021 (2)	0.0006 (17)	0.0056 (19)	0.0014 (17)
C19	0.011 (2)	0.017 (2)	0.023 (2)	0.0020 (18)	−0.0004 (19)	0.0108 (19)
C20	0.018 (2)	0.024 (3)	0.017 (2)	−0.004 (2)	−0.004 (2)	0.009 (2)
C21	0.017 (2)	0.016 (2)	0.014 (2)	−0.0025 (19)	−0.0036 (19)	0.0015 (18)
C22	0.013 (2)	0.024 (3)	0.0084 (19)	−0.002 (2)	−0.0013 (17)	−0.0025 (18)
C23	0.010 (2)	0.013 (2)	0.014 (2)	−0.0029 (17)	−0.0014 (18)	−0.0018 (17)
C24	0.009 (2)	0.012 (2)	0.014 (2)	0.0035 (16)	0.0032 (17)	−0.0014 (16)
C25	0.0072 (19)	0.012 (2)	0.016 (2)	0.0006 (16)	0.0023 (18)	−0.0046 (17)
C26	0.013 (2)	0.0080 (19)	0.0137 (19)	−0.0034 (17)	−0.0007 (17)	0.0005 (16)
C27	0.021 (2)	0.014 (2)	0.014 (2)	−0.0050 (19)	0.002 (2)	−0.0065 (17)
C28	0.013 (2)	0.0046 (19)	0.017 (2)	0.0021 (16)	0.0002 (19)	−0.0015 (16)
O1	0.0051 (14)	0.0118 (15)	0.0094 (14)	0.0019 (12)	0.0024 (12)	−0.0015 (11)
O2	0.0050 (14)	0.0105 (15)	0.0149 (15)	−0.0010 (12)	0.0052 (12)	0.0019 (12)
O3	0.0074 (15)	0.0087 (14)	0.0120 (14)	0.0004 (11)	−0.0032 (12)	0.0046 (11)
O4	0.0066 (15)	0.0113 (15)	0.0125 (15)	−0.0018 (12)	0.0003 (12)	−0.0040 (12)
O5	0.0037 (14)	0.0078 (14)	0.0173 (16)	0.0010 (11)	−0.0014 (13)	−0.0021 (12)
O6	0.0075 (14)	0.0103 (15)	0.0151 (15)	0.0015 (12)	0.0024 (13)	−0.0031 (12)
C29	0.0080 (17)	0.0044 (16)	0.0079 (17)	−0.0027 (14)	−0.0010 (14)	−0.0048 (13)
C30	0.0039 (16)	0.0064 (17)	0.0072 (16)	−0.0001 (14)	−0.0003 (14)	−0.0013 (13)
C31	0.0041 (16)	0.0086 (17)	0.0075 (16)	0.0002 (14)	0.0020 (14)	0.0008 (13)
C32	0.0060 (16)	0.0057 (16)	0.0071 (16)	0.0006 (14)	−0.0001 (14)	0.0038 (13)
O7	0.0047 (13)	0.0089 (13)	0.0129 (14)	−0.0003 (11)	−0.0039 (12)	0.0004 (11)
O8	0.0078 (14)	0.0091 (14)	0.0136 (14)	0.0020 (11)	−0.0024 (12)	0.0010 (11)
O9	0.0076 (14)	0.0114 (14)	0.0114 (13)	−0.0010 (11)	0.0040 (12)	0.0047 (11)
O10	0.0055 (13)	0.0105 (14)	0.0128 (14)	0.0017 (11)	0.0027 (12)	−0.0062 (11)
O11	0.0048 (13)	0.0066 (13)	0.0118 (14)	0.0012 (11)	−0.0025 (11)	−0.0006 (11)
O12	0.0101 (14)	0.0095 (14)	0.0138 (14)	−0.0018 (12)	−0.0016 (12)	−0.0045 (12)
C33	0.0086 (17)	0.0043 (16)	0.0091 (16)	0.0023 (14)	−0.0018 (15)	−0.0036 (14)
C34	0.0046 (16)	0.0068 (17)	0.0070 (16)	−0.0008 (13)	0.0014 (14)	−0.0018 (14)
C35	0.0009 (15)	0.0065 (17)	0.0102 (16)	0.0008 (13)	−0.0017 (14)	0.0008 (13)
C36	0.0070 (17)	0.0081 (17)	0.0047 (15)	−0.0004 (14)	−0.0023 (14)	0.0004 (13)
O1W	0.0233 (19)	0.0127 (16)	0.0170 (16)	0.0060 (15)	−0.0065 (15)	−0.0027 (13)
O2W	0.0172 (17)	0.0093 (15)	0.0255 (18)	−0.0048 (14)	−0.0002 (15)	−0.0022 (13)
O3W	0.0138 (17)	0.0119 (16)	0.0227 (17)	0.0042 (13)	−0.0016 (15)	−0.0046 (13)
O4W	0.0082 (15)	0.0123 (15)	0.0095 (14)	0.0018 (13)	−0.0012 (12)	−0.0019 (11)
O5W	0.0075 (14)	0.0099 (14)	0.0098 (14)	−0.0026 (12)	−0.0003 (12)	0.0039 (11)
O6W	0.0089 (15)	0.0136 (16)	0.0113 (14)	−0.0029 (13)	−0.0001 (13)	−0.0055 (12)
O7W	0.0187 (18)	0.0106 (15)	0.0137 (15)	−0.0071 (14)	0.0046 (14)	−0.0016 (12)
O8W	0.0131 (16)	0.0117 (16)	0.0211 (17)	0.0013 (13)	0.0070 (14)	0.0005 (14)
O9W	0.0070 (15)	0.0163 (16)	0.0082 (14)	0.0030 (12)	0.0024 (12)	0.0041 (12)

### Geometric parameters (Å, °)

**Table:**

N1—C1	1.324 (5)	C24—C28	1.503 (6)
N1—C11	1.354 (5)	C25—C26	1.447 (6)
N2—C10	1.338 (6)	C27—H27A	0.9800
N2—C12	1.355 (5)	C27—H27B	0.9800
N2—H2A	0.8800	C27—H27C	0.9800
C1—C2	1.419 (6)	C28—H28A	0.9800
C1—C13	1.494 (6)	C28—H28B	0.9800
C2—C3	1.369 (6)	C28—H28C	0.9800
C2—H2	0.9500	O1—C29	1.307 (5)
C3—C4	1.426 (6)	O1—H1	0.99 (6)
C3—H3	0.9500	O2—C29	1.217 (5)
C4—C11	1.398 (6)	O3—C30	1.422 (5)
C4—C5	1.434 (6)	O3—H3B	0.8400
C5—C6	1.355 (7)	O4—C31	1.418 (5)
C5—H5	0.9500	O4—H4	0.8400
C6—C7	1.438 (6)	O5—C32	1.294 (5)
C6—H6	0.9500	O6—C32	1.236 (5)
C7—C12	1.406 (6)	C29—C30	1.529 (6)
C7—C8	1.415 (6)	C30—C31	1.524 (6)
C8—C9	1.371 (7)	C30—H30	1.0000
C8—H8	0.9500	C31—C32	1.530 (6)
C9—C10	1.399 (6)	C31—H31	1.0000
C9—H9	0.9500	O7—C33	1.309 (5)
C10—C14	1.503 (6)	O7—H7	0.91 (6)
C11—C12	1.445 (6)	O8—C33	1.219 (5)
C13—H13A	0.9800	O9—C34	1.412 (5)
C13—H13B	0.9800	O9—H9C	0.8400
C13—H13C	0.9800	O10—C35	1.411 (5)
C14—H14A	0.9800	O10—H10A	0.8400
C14—H14B	0.9800	O11—C36	1.287 (5)
C14—H14C	0.9800	O12—C36	1.230 (5)
N3—C15	1.335 (6)	C33—C34	1.524 (6)
N3—C25	1.356 (6)	C34—C35	1.542 (6)
N4—C24	1.340 (5)	C34—H34	1.0000
N4—C26	1.360 (6)	C35—C36	1.530 (6)
N4—H4C	0.8800	C35—H35	1.0000
C15—C16	1.431 (6)	O1W—H1A	0.87 (4)
C15—C27	1.486 (7)	O1W—H1B	0.87 (4)
C16—C17	1.371 (7)	O2W—H2C	0.86 (4)
C16—H16	0.9500	O2W—H2D	0.88
C17—C18	1.414 (6)	O3W—H3C	0.87 (4)
C17—H17	0.9500	O3W—H3D	0.87 (4)
C18—C25	1.405 (6)	O4W—H4A	0.87 (4)
C18—C19	1.442 (7)	O4W—H4B	0.87 (4)
C19—C20	1.344 (7)	O5W—H5A	0.87 (4)
C19—H19	0.9500	O5W—H5B	0.87 (4)
C20—C21	1.436 (7)	O6W—H6A	0.87 (4)
C20—H20	0.9500	O6W—H6B	0.86 (3)
C21—C26	1.400 (6)	O7W—H7A	0.87 (4)
C21—C22	1.421 (7)	O7W—H7B	0.87 (4)
C22—C23	1.356 (7)	O8W—H8A	0.87 (4)
C22—H22	0.9500	O8W—H8B	0.87 (2)
C23—C24	1.405 (6)	O9W—H9A	0.86 (4)
C23—H23	0.9500	O9W—H9B	0.87 (4)
			
C1—N1—C11	117.2 (4)	C23—C22—H22	119.5
C10—N2—C12	123.5 (4)	C21—C22—H22	119.5
C10—N2—H2A	118.3	C22—C23—C24	120.2 (4)
C12—N2—H2A	118.3	C22—C23—H23	119.9
N1—C1—C2	122.3 (4)	C24—C23—H23	119.9
N1—C1—C13	116.6 (4)	N4—C24—C23	118.6 (4)
C2—C1—C13	121.1 (4)	N4—C24—C28	119.1 (4)
C3—C2—C1	120.3 (4)	C23—C24—C28	122.3 (4)
C3—C2—H2	119.9	N3—C25—C18	125.1 (4)
C1—C2—H2	119.9	N3—C25—C26	116.8 (4)
C2—C3—C4	118.5 (4)	C18—C25—C26	118.0 (4)
C2—C3—H3	120.8	N4—C26—C21	119.9 (4)
C4—C3—H3	120.8	N4—C26—C25	118.8 (4)
C11—C4—C3	116.5 (4)	C21—C26—C25	121.3 (4)
C11—C4—C5	121.0 (4)	C15—C27—H27A	109.5
C3—C4—C5	122.6 (4)	C15—C27—H27B	109.5
C6—C5—C4	120.4 (4)	H27A—C27—H27B	109.5
C6—C5—H5	119.8	C15—C27—H27C	109.5
C4—C5—H5	119.8	H27A—C27—H27C	109.5
C5—C6—C7	120.7 (4)	H27B—C27—H27C	109.5
C5—C6—H6	119.6	C24—C28—H28A	109.5
C7—C6—H6	119.6	C24—C28—H28B	109.5
C12—C7—C8	117.4 (4)	H28A—C28—H28B	109.5
C12—C7—C6	119.3 (4)	C24—C28—H28C	109.5
C8—C7—C6	123.3 (4)	H28A—C28—H28C	109.5
C9—C8—C7	120.4 (4)	H28B—C28—H28C	109.5
C9—C8—H8	119.8	C29—O1—H1	116 (3)
C7—C8—H8	119.8	C30—O3—H3B	109.5
C8—C9—C10	120.3 (4)	C31—O4—H4	109.5
C8—C9—H9	119.9	O2—C29—O1	125.0 (4)
C10—C9—H9	119.9	O2—C29—C30	121.9 (4)
N2—C10—C9	118.6 (4)	O1—C29—C30	113.0 (4)
N2—C10—C14	119.2 (4)	O3—C30—C31	111.2 (3)
C9—C10—C14	122.2 (4)	O3—C30—C29	108.9 (3)
N1—C11—C4	125.1 (4)	C31—C30—C29	111.0 (4)
N1—C11—C12	116.7 (4)	O3—C30—H30	108.6
C4—C11—C12	118.1 (4)	C31—C30—H30	108.6
N2—C12—C7	119.8 (4)	C29—C30—H30	108.6
N2—C12—C11	119.8 (4)	O4—C31—C30	111.4 (3)
C7—C12—C11	120.4 (4)	O4—C31—C32	109.1 (3)
C1—C13—H13A	109.5	C30—C31—C32	110.9 (4)
C1—C13—H13B	109.5	O4—C31—H31	108.5
H13A—C13—H13B	109.5	C30—C31—H31	108.5
C1—C13—H13C	109.5	C32—C31—H31	108.5
H13A—C13—H13C	109.5	O6—C32—O5	124.0 (4)
H13B—C13—H13C	109.5	O6—C32—C31	120.9 (4)
C10—C14—H14A	109.5	O5—C32—C31	115.1 (4)
C10—C14—H14B	109.5	C33—O7—H7	111 (3)
H14A—C14—H14B	109.5	C34—O9—H9C	109.5
C10—C14—H14C	109.5	C35—O10—H10A	109.5
H14A—C14—H14C	109.5	O8—C33—O7	125.2 (4)
H14B—C14—H14C	109.5	O8—C33—C34	121.6 (4)
C15—N3—C25	117.1 (4)	O7—C33—C34	113.2 (4)
C24—N4—C26	123.2 (4)	O9—C34—C33	108.6 (4)
C24—N4—H4C	118.4	O9—C34—C35	111.5 (3)
C26—N4—H4C	118.4	C33—C34—C35	111.3 (4)
N3—C15—C16	121.8 (4)	O9—C34—H34	108.5
N3—C15—C27	116.9 (4)	C33—C34—H34	108.5
C16—C15—C27	121.3 (4)	C35—C34—H34	108.5
C17—C16—C15	120.6 (4)	O10—C35—C36	109.6 (4)
C17—C16—H16	119.7	O10—C35—C34	111.2 (3)
C15—C16—H16	119.7	C36—C35—C34	109.7 (4)
C16—C17—C18	118.3 (4)	O10—C35—H35	108.8
C16—C17—H17	120.8	C36—C35—H35	108.8
C18—C17—H17	120.8	C34—C35—H35	108.8
C25—C18—C17	117.1 (4)	O12—C36—O11	125.4 (4)
C25—C18—C19	119.7 (4)	O12—C36—C35	119.4 (4)
C17—C18—C19	123.2 (4)	O11—C36—C35	115.1 (4)
C20—C19—C18	121.4 (4)	H1A—O1W—H1B	103 (4)
C20—C19—H19	119.3	H2C—O2W—H2D	103
C18—C19—H19	119.3	H3C—O3W—H3D	102 (4)
C19—C20—C21	121.0 (5)	H4A—O4W—H4B	101 (4)
C19—C20—H20	119.5	H5A—O5W—H5B	101 (3)
C21—C20—H20	119.5	H6A—O6W—H6B	105 (4)
C26—C21—C22	117.1 (4)	H7A—O7W—H7B	102 (4)
C26—C21—C20	118.6 (4)	H8A—O8W—H8B	98 (4)
C22—C21—C20	124.2 (4)	H9A—O9W—H9B	102 (4)
C23—C22—C21	121.0 (4)		
			
C11—N1—C1—C2	2.7 (7)	C26—C21—C22—C23	0.0 (7)
C11—N1—C1—C13	−175.5 (4)	C20—C21—C22—C23	177.4 (5)
N1—C1—C2—C3	−2.3 (8)	C21—C22—C23—C24	0.4 (7)
C13—C1—C2—C3	175.8 (5)	C26—N4—C24—C23	−1.6 (7)
C1—C2—C3—C4	−0.2 (7)	C26—N4—C24—C28	178.9 (4)
C2—C3—C4—C11	2.1 (7)	C22—C23—C24—N4	0.4 (7)
C2—C3—C4—C5	−177.6 (5)	C22—C23—C24—C28	179.9 (5)
C11—C4—C5—C6	−3.0 (7)	C15—N3—C25—C18	1.0 (7)
C3—C4—C5—C6	176.6 (5)	C15—N3—C25—C26	178.4 (4)
C4—C5—C6—C7	3.3 (7)	C17—C18—C25—N3	−1.6 (7)
C5—C6—C7—C12	−0.5 (7)	C19—C18—C25—N3	177.7 (4)
C5—C6—C7—C8	−179.4 (5)	C17—C18—C25—C26	−179.0 (4)
C12—C7—C8—C9	3.1 (7)	C19—C18—C25—C26	0.3 (7)
C6—C7—C8—C9	−177.9 (5)	C24—N4—C26—C21	2.0 (7)
C7—C8—C9—C10	−1.4 (8)	C24—N4—C26—C25	−176.6 (4)
C12—N2—C10—C9	0.9 (7)	C22—C21—C26—N4	−1.1 (7)
C12—N2—C10—C14	−179.4 (4)	C20—C21—C26—N4	−178.7 (5)
C8—C9—C10—N2	−0.7 (7)	C22—C21—C26—C25	177.4 (5)
C8—C9—C10—C14	179.6 (5)	C20—C21—C26—C25	−0.1 (7)
C1—N1—C11—C4	−0.6 (7)	N3—C25—C26—N4	1.1 (6)
C1—N1—C11—C12	177.5 (4)	C18—C25—C26—N4	178.7 (4)
C3—C4—C11—N1	−1.8 (7)	N3—C25—C26—C21	−177.5 (4)
C5—C4—C11—N1	177.9 (5)	C18—C25—C26—C21	0.1 (7)
C3—C4—C11—C12	−179.9 (4)	O2—C29—C30—O3	11.4 (6)
C5—C4—C11—C12	−0.2 (7)	O1—C29—C30—O3	−169.4 (3)
C10—N2—C12—C7	1.0 (7)	O2—C29—C30—C31	134.1 (4)
C10—N2—C12—C11	−178.2 (4)	O1—C29—C30—C31	−46.7 (5)
C8—C7—C12—N2	−2.9 (7)	O3—C30—C31—O4	60.1 (4)
C6—C7—C12—N2	178.1 (4)	C29—C30—C31—O4	−61.2 (5)
C8—C7—C12—C11	176.2 (4)	O3—C30—C31—C32	−61.5 (5)
C6—C7—C12—C11	−2.8 (7)	C29—C30—C31—C32	177.1 (3)
N1—C11—C12—N2	4.0 (6)	O4—C31—C32—O6	4.8 (6)
C4—C11—C12—N2	−177.8 (4)	C30—C31—C32—O6	127.8 (4)
N1—C11—C12—C7	−175.2 (4)	O4—C31—C32—O5	−176.0 (3)
C4—C11—C12—C7	3.0 (7)	C30—C31—C32—O5	−53.1 (5)
C25—N3—C15—C16	0.3 (6)	O8—C33—C34—O9	13.3 (6)
C25—N3—C15—C27	179.7 (4)	O7—C33—C34—O9	−168.2 (3)
N3—C15—C16—C17	−1.0 (7)	O8—C33—C34—C35	136.4 (4)
C27—C15—C16—C17	179.7 (5)	O7—C33—C34—C35	−45.1 (5)
C15—C16—C17—C18	0.4 (7)	O9—C34—C35—O10	58.9 (4)
C16—C17—C18—C25	0.8 (7)	C33—C34—C35—O10	−62.5 (5)
C16—C17—C18—C19	−178.4 (5)	O9—C34—C35—C36	−62.5 (5)
C25—C18—C19—C20	−0.6 (7)	C33—C34—C35—C36	176.1 (3)
C17—C18—C19—C20	178.6 (5)	O10—C35—C36—O12	1.5 (6)
C18—C19—C20—C21	0.5 (8)	C34—C35—C36—O12	123.8 (4)
C19—C20—C21—C26	−0.2 (8)	O10—C35—C36—O11	179.6 (3)
C19—C20—C21—C22	−177.5 (5)	C34—C35—C36—O11	−58.1 (5)

### Hydrogen-bond geometry (Å, °)

**Table:**

*D*—H···*A*	*D*—H	H···*A*	*D*···*A*	*D*—H···*A*
N2—H2A···O1W	0.88	1.97	2.820 (5)	162.
N4—H4C···O7W	0.88	1.98	2.814 (5)	159.
O4—H4···O6W	0.84	1.80	2.633 (5)	175.
O9—H9C···O5W	0.84	1.83	2.673 (4)	177.
O10—H10A···O4W	0.84	1.79	2.627 (5)	172.
O2W—H2C···O11	0.86 (4)	1.97 (2)	2.783 (4)	157 (4)
O3W—H3C···O5	0.87 (4)	1.95 (2)	2.798 (5)	168 (5)
O2W—H2D···O8W	0.88	2.42	2.903 (5)	115
O5W—H5A···O3	0.87 (4)	2.04 (3)	2.827 (4)	151 (5)
O5W—H5A···O2	0.87 (4)	2.35 (4)	3.020 (4)	135 (5)
O8W—H8B···O2W	0.87 (2)	2.03 (2)	2.903 (5)	178 (5)
O9W—H9A···O2	0.86 (4)	1.89 (2)	2.744 (4)	175 (5)
O9W—H9B···O9	0.87 (4)	2.08 (4)	2.849 (4)	147 (5)
O9W—H9B···O8	0.87 (4)	2.26 (4)	2.958 (4)	137 (5)
O1—H1···O5^i^	0.99 (6)	1.49 (6)	2.473 (4)	172 (5)
O7—H7···O11^i^	0.91 (6)	1.60 (6)	2.496 (4)	167 (5)
O4W—H4B···O12^i^	0.87 (2)	1.85 (2)	2.720 (5)	178 (6)
O6W—H6B···O6^i^	0.86 (3)	1.93 (2)	2.776 (5)	167 (5)
O3—H3B···O9W^ii^	0.84	1.81	2.647 (5)	173.
O5W—H5B···O8^ii^	0.87 (4)	1.91 (2)	2.761 (4)	168 (5)
O1W—H1A···O2W^iii^	0.87 (4)	2.22 (3)	3.062 (5)	161 (5)
O1W—H1B···O5W^iii^	0.87 (4)	1.96 (2)	2.811 (5)	168 (6)
O3W—H3D···O8W^iii^	0.87 (4)	2.02 (2)	2.876 (5)	168 (5)
O4W—H4A···O10^iv^	0.87 (4)	1.99 (3)	2.815 (4)	160 (5)
O4W—H4A···O12^iv^	0.87 (4)	2.40 (4)	2.998 (4)	127 (4)
O6W—H6A···O4^v^	0.87 (4)	2.15 (4)	2.892 (4)	143 (5)
O6W—H6A···O6^v^	0.87 (4)	2.19 (4)	2.909 (5)	140 (5)
O7W—H7A···O3W^vi^	0.87 (4)	2.17 (3)	2.988 (5)	156 (5)
O7W—H7B···O9W^vii^	0.87 (4)	2.02 (2)	2.869 (5)	165 (5)
O8W—H8A···O1^vii^	0.87 (4)	2.06 (2)	2.926 (5)	170 (5)

Symmetry codes: (i) *x*−1, *y*, *z*; (ii) *x*+1, *y*, *z*; (iii) −*x*, *y*+1/2, −*z*+1/2; (iv) *x*−1/2, −*y*+1/2, −*z*+1; (v) *x*−1/2, −*y*+1/2, −*z*; (vi) −*x*+1, *y*−1/2, −*z*+1/2; (vii) −*x*, *y*−1/2, −*z*+1/2.
